# Factors Associated with Outcomes of Patients with Veno-Venous Extracorporeal Membrane Oxygenation for COVID-19

**DOI:** 10.3390/jcm13195922

**Published:** 2024-10-04

**Authors:** Soojin Lee, Gayeon Kang, Seunghwan Song, Kwangha Lee, Wanho Yoo, Hyojin Jang, Myung Hun Jang

**Affiliations:** 1 Department of Thoracic and Cardiovascular Surgery, Pusan National University School of Medicine, Biomedical Research Institute, Pusan National University Hospital, Busan 49241, Republic of Korea; lsj31514401@gmail.com (S.L.); gayeon0916@naver.com (G.K.); 2 Division of Pulmonary, Allergy, and Critical Care Medicine, Biomedical Research Institute, Department of Internal Medicine, Pusan National University Hospital, Busan 49241, Republic of Korea; jubilate@pusan.ac.kr (K.L.); lcc2202@naver.com (W.Y.); hyoding2@naver.com (H.J.); 3 Department of Rehabilitation Medicine, Biomedical Research Institute, Pusan National University Hospital, Busan 49241, Republic of Korea; zmh1048@naver.com

**Keywords:** ECMO, COVID-19, ARDS

## Abstract

**Background:** The World Health Organization recommends extracorporeal membrane oxygenation (ECMO) as a therapeutic option for the most critical cases of severe coronavirus disease 2019 (COVID-19). However, data on universally agreed-upon risk factors that contribute to ECMO weaning failure and mortality in COVID-19 patients undergoing ECMO are limited. This lack of consensus leads to significant uncertainties in developing effective management strategies for these patients. We aimed to identify the factors associated with early outcomes after ECMO support in patients with COVID-19-induced acute respiratory distress syndrome, specifically the success rate of ECMO weaning and in-hospital mortality. **Methods:** We reviewed 25 patients with COVID-19 who received ECMO support at a single institution between January 2020 and July 2022. This retrospective data collection and review included clinical characteristics, adjunctive treatments, complications, and early patient outcomes. **Results:** A total of 72% of the patients were successfully weaned off ECMO, and 68% were discharged alive. Significant associations were observed between ECMO weaning success and in-hospital survival, particularly younger age and a history of rehabilitation therapy. Furthermore, the absence of a history of immunosuppressive therapy contributed significantly to successful ECMO weaning. **Conclusions:** Younger age and the implementation of rehabilitation therapy are associated with improved outcomes in patients with COVID-19 receiving ECMO support.

## 1. Introduction

In March 2020, the World Health Organization (WHO) declared coronavirus disease 2019 (COVID-19), caused by the Severe Acute Respiratory Syndrome Coronavirus 2 (SARS-CoV-2), a global pandemic [1]. Managing COVID-19, particularly its severe and protracted progression to acute respiratory distress syndrome (ARDS), presents significant challenges for intensive care practitioners [2]. The WHO recommends extracorporeal membrane oxygenation (ECMO) as a therapeutic option for the most critical cases of severe COVID-19 [3]. Although ECMO can be life-saving and aid in recovery, it requires substantial resources and may lead to severe complications [2]. Preliminary evidence indicates a possible advantage of ECMO in selected patients with ARDS associated with COVID-19, although the outcomes are highly contingent upon the criteria for patient selection and the timing of ECMO initiation [4]. Despite the ongoing pandemic, data on COVID-19 patients undergoing ECMO remain limited, particularly concerning significant risk factors that are universally agreed upon as contributing to ECMO weaning failure and mortality. This lack of consensus adds to the uncertainties in effectively managing these patients [5,6,7,8]. Critical decisions regarding ECMO, particularly in resource-limited settings, are challenging for clinicians.

We aimed to elucidate the characteristics and implications of ECMO therapy in managing ARDS induced by COVID-19 and to identify both structural and patient-specific factors that influence the outcomes of ECMO treatment in patients with COVID-19.

## 2. Materials and Methods

### 2.1. Ethical Statement

This observational clinical study did not require clinical trial registration. It involved the review of deidentified medical records to ensure patient anonymity. This study was approved by the Institutional Review Board of Pusan National University Hospital (IRB No. B-2302-014-124). The requirement for informed consent was waived because the analysis was conducted retrospectively using electronic medical records. Images of patients used in this study are under explicit written consent, with all identifiable features appropriately anonymized to protect patient privacy. The consent form clarified the use of these images in academic publications and was duly signed by the patients involved.

### 2.2. Study Design and Patient Population

**Figure 1:**
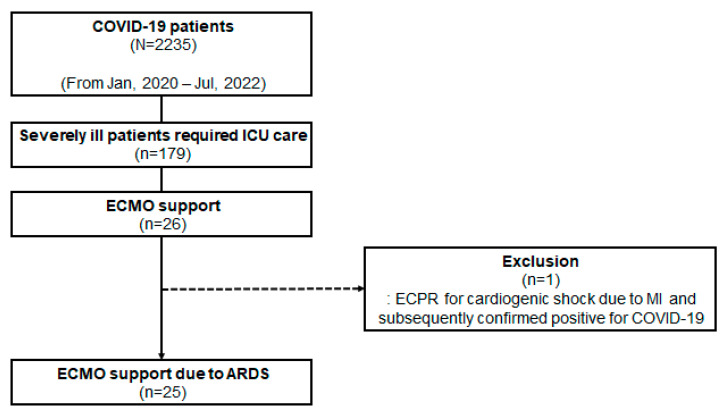
Study flowchart. ICU, intensive care unit; ECMO, extracorporeal membrane oxygenation; ECPR, extracorporeal cardiopulmonary resuscitation; MI, myocardial infarction; ARDS, acute respiratory distress syndrome.

Between January 2020 and July 2022, our center diagnosed 2235 patients with COVID-19 by real-time reverse transcriptase polymerase chain reaction testing. Among these individuals, 179 required treatment in the intensive care unit (ICU) due to severe illness, with 26 receiving ECMO therapy. One patient who underwent ECMO as part of extracorporeal cardiopulmonary resuscitation for cardiogenic shock was excluded from the analysis. Consequently, this study focused on 25 patients who underwent veno-venous ECMO for ARDS (Figure 1).

Patients who underwent ECMO support were stratified into two cohorts based on the outcome of ECMO weaning: those who were successfully weaned off ECMO (Weaning Success Group, *n* = 18) and those in whom weaning was unsuccessful (Weaning Failure Group, *n* = 7). This stratification was used to identify and analyze the predictors associated with successful weaning from ECMO.

Furthermore, an assessment of in-hospital mortality categorized patients into two groups: individuals who survived until discharge (Discharge Group, *n* = 17) and those who died in hospital (Death Group, *n* = 8). This classification enabled a detailed examination of the factors associated with in-hospital mortality in the ECMO patient population.

### 2.3. Analyzed Factors

The data collection process for this investigation encompassed various aspects such as patient characteristics and pre- and post-ECMO support parameters. Records were maintained for patient demographics and clinical profiles, including sex, age, pre-existing conditions, smoking history, and body mass index (BMI). The study also gathered data on adjunctive respiratory treatments, including the administration of antiviral medications, immunosuppressants, steroids, prone positioning, inotropics, vasopressors, and neuromuscular blocking agents. We documented information on the settings and duration of mechanical ventilation, provision of rehabilitation therapy, and laboratory results as parameters before and after the initiation of ECMO support. Furthermore, additional details concerning ECMO outcomes, such as the duration of ECMO support, complications encountered, successful weaning from ECMO, and in-hospital mortality rates, were systematically collected.

### 2.4. The COVID-19 Treatment Strategy at Our Center

In our treatment protocol for confirmed SARS-CoV-2 infection, patients requiring noninvasive oxygen support received a ten-day course of remdesivir (Gilead Sciences, Inc., Foster City, CA, USA), an antiviral compound [9]. Anticipatory antibiotic treatment is initiated in cases of suspected pneumonia prior to COVID-19 confirmation or when postdiagnosis conditions necessitate intubation, incorporating a regimen of fourth-generation cephalosporins, penicillin, and fluoroquinolones [10].

Simultaneously, for those who required supplemental oxygen, corticosteroid therapy with dexamethasone (6 mg/day intravenously) was commenced for ten days and extended based on clinical evaluation, regardless of microbial culture findings [11]. In cases of clinical exacerbation despite corticosteroid therapy, the protocol included the administration of tocilizumab (ACTEMRA; Genentech, South San Francisco, CA, USA), an immunosuppressive agent [12]. Fluconazole (JW Pharmaceutical, Seoul, South Korea) was prescribed concurrently as a prophylactic agent against secondary fungal infections.

Moreover, the initiation of mechanical ventilation is accompanied by the prophylactic administration of low-molecular-weight heparin to prevent deep vein thrombosis and H2 receptor antagonists to mitigate the risk of stress-induced gastric ulcers.

### 2.5. Indication of Veno-Venous ECMO for Patients with COVID-19

The criteria for initiating veno-venous ECMO in patients with COVID-19 adhered to the guidelines established by the Extracorporeal Life Support Organization (ELSO). Indications for ECMO initiation included a partial pressure of arterial oxygen to fractional inspired oxygen (PaO_2_/FiO_2_ or P/F) ratio below 80 mmHg persisting for more than 6 h, a P/F ratio below 50 mmHg for more than 3 h, or arterial blood pH below 7.25 with a partial pressure of carbon dioxide (PaCO_2_) equal to or exceeding 60 mmHg for a duration exceeding 6 h [13].

Relative contraindications included prolonged mechanical ventilation (more than seven days) before ECMO, advanced age, history of cardiac arrest, significant comorbid conditions, and extrapulmonary organ failure. However, in circumstances where both the patient and their family supported aggressive management and ECMO represented the sole therapeutic option, its application might be considered following a comprehensive discussion among a multidisciplinary team.

Cannulation procedures were performed exclusively in an isolated ward setting using a percutaneous technique under ultrasound guidance for accuracy. The veno-venous approach primarily involved inserting a drainage cannula into the femoral vein and a return cannula into the right internal jugular vein. The determination of the cannula size for veno-venous ECMO was multifactorial, considering the patient’s body surface area, vascular caliber as assessed by computed tomography scans and preprocedural ultrasound, and the expertise of the cardiovascular surgical team. The cannula sizes utilized ranged from 12 to 24 French.

### 2.6. Anticoagulation Methods during ECMO Support

Heparin was the anticoagulant of choice throughout the ECMO. Heparin (doses ranging from 50 to 100 IU/kg) was administered intravenously just before cannulation, except in patients with bleeding complications. Anticoagulation effects were monitored and adjusted concurrently using activated clotting time (ACT) and activated partial thromboplastin time. The target ACT was maintained within a therapeutic range of 150–200 s, ensuring optimal anticoagulation while minimizing bleeding risk [14].

In cases where HIT is suspected and other causes of thrombocytopenia are ruled out, our center discontinues heparin and initiates a direct thrombin inhibitor, specifically Argatroban (Mitsubishi Tanabe Pharma Corporation, Osaka, Japan). The efficacy is monitored using ACT and PTT, with the dosage titrated to achieve a clotting time 1.5 times the normal range, similar to our heparin protocol [14].

### 2.7. Rehabilitation Program

Rehabilitation interventions commenced once patients exhibited stable vital signs and were capable of co-operation under mild sedation, as indicated by a Richmond Agitation–Sedation Scale (RASS) score of −2. The decision to proceed with daily rehabilitation activities was made after evaluating the patient’s condition. Muscle strength was assessed using the Medical Research Council Sum Score (MRC-SS), a commonly used tool for evaluating muscle strength in ICU patients. This tool has been routinely employed at our center, and the rehabilitation stage for each patient was determined based on their individual strength assessment. Primary rehabilitation efforts focused on enhancing strength in the upper and lower extremities and respiratory function, facilitated by positioning the patient with an elevated head. If a catheter was placed in the femoral vessel, hip flexion was limited to within 30°. The rehabilitation regimen was structured to include joint movements in the neck, shoulder, forearm, wrist, hip, knee, and ankle areas. Exercise-based interventions were conducted three times weekly for the first two weeks, followed by once-weekly sessions for the subsequent two weeks.

**Figure 2:**
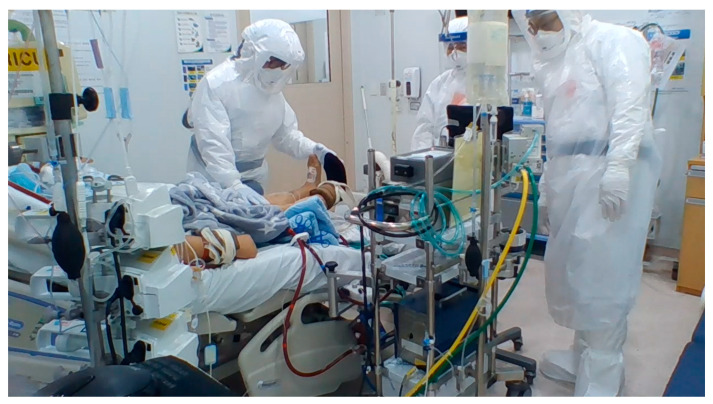
Photograph of a patient undergoing rehabilitation therapy in an isolation ward with ECMO support for COVID-19. An awake patient on veno-venous ECMO support is undergoing lower limb rehabilitation therapy. Before starting the therapy, the range of leg movement was assessed to ensure the ECMO cannula had sufficient length to prevent dislodgement, and the ECMO line was secured to the right side of the bed. The cannula inserted into the patient’s right neck was further stabilized with a bandage around the forehead, and the patient was instructed to minimize upper body movement. The femoral cannula was held securely during the rehabilitation exercise to maintain its position.

To mitigate the risk of accidental decannulation of the ECMO device, rehabilitation sessions were overseen by physicians from the Department of Rehabilitation, with continuous monitoring of the patient’s vital signs and ECMO flow dynamics by the ECMO team. The ECMO team included cardiovascular surgeons, ICU intensivists, and perfusionists, ensuring a multidisciplinary approach to patient care during rehabilitation. Patients undergoing femoral cannulation were advised to practice daily activities, including standing outside the bed, facilitated by an interdisciplinary ECMO team. Weaning from ECMO and decannulation were conducted at various stages of rehabilitation therapy, from bedbound to standing positions (Figure 2).

### 2.8. Statistical Analysis

The associations between various factors and outcomes related to ECMO weaning success and mortality rates were investigated. For the analysis of continuous variables, either the independent *t*-test or the Wilcoxon rank-sum test was employed based on the data distribution. Categorical variables were analyzed using either the chi-squared test or Fisher’s exact test, depending on the expected frequencies in the contingency tables. Data are presented as mean ± standard deviation (SD) for normally distributed variables, counts and percentages for categorical variables, or as median and interquartile range for non-normally distributed continuous variables. Statistical analyses were performed using Statistical Package for the Social Sciences (SPSS) software version 22. Statistical significance was set at *p* < 0.05.

## 3. Results

### 3.1. Baseline Patient Characteristics

Table 1 presents the baseline characteristics of the study population, including 25 patients who received ECMO support for COVID-19 treatment. The mean Sequential Organ Failure Assessment (SOFA) score among these patients was 5.6 (±1.83), indicating the severity of organ dysfunction at ECMO initiation. The mean age of the cohort was 51 years (±15.38), and the average duration of ECMO support was 14.88 days (±10.05), highlighting the variability in the length of ECMO support required. Supplementary materials contain additional findings related to the comorbidities associated with ECMO weaning success and in-hospital mortality among COVID-19 patients.

### 3.2. Factors Associated with ECMO Weaning Success of Patients with COVID-19

The ECMO weaning success rate in our study was 72% (*n* = 17), while the ECMO failure rate was 28% (*n* = 8).

Table 2 presents a comparison of perioperative factors between the ECMO weaning success and weaning failure groups. The analysis revealed a statistically significant younger mean age within the ECMO Weaning Success Group (46.78 ± 15.06 years) compared to the ECMO Weaning Failure Group (65 ± 5.03 years) (*p* < 0.001). Participation in rehabilitation therapy during ECMO support was markedly higher in the Weaning Success Group (16 of 18 patients, 88.9%) compared to the Weaning Failure Group (1 of 7 patients, 14.3%), demonstrating a statistically significant association (*p* < 0.001).

In contrast, the presence of a history of immunosuppressive therapy during admission was significantly more prevalent in the ECMO Weaning Failure Group (6 of 7 patients, 85.7%) than in the Weaning Success Group (6 of 18 patients, 33.3%), with the difference being statistically significant (*p* = 0.030). Subsequent correlation analysis of patients who developed secondary infections after immunosuppressive treatment indicated that the occurrence of secondary infections did not significantly differ between the ECMO weaning success and ECMO weaning failure groups (Table 3).

### 3.3. Factors Associated with In-Hospital Mortality of Patients with COVID-19

The survival-to-discharge rate observed in our study was 68% (n = 17), whereas the in-hospital mortality rate accounted for 32% (n = 8).

Table 4 presents a comparative analysis of perioperative factors between the groups defined by survival-to-discharge and in-hospital mortality. The analysis indicated a significantly lower mean age in the Discharge Group (45.38 ± 15.32 years) as opposed to the Death Group (63.44 ± 5.90 years), with the difference being statistically significant (*p* < 0.001). Furthermore, the engagement in rehabilitation therapy during ECMO support was substantially more frequent in the Discharge Group (16 of 17 patients, 94.1%) in comparison to the Death Group (0 of 8 patients, 0%), which also yielded a statistically significant correlation (*p* < 0.001).

## 4. Discussion

The use of veno-venous ECMO significantly increased during the COVID-19 pandemic, with initial investigations suggesting a favorable prognosis in terms of survival rates [15,16]. Early outcomes for patients with COVID-19 at our institution treated with ECMO showed promising results, with high rates of weaning and discharge survival, matching or even surpassing the findings from other sources, such as the ELSO Registry and studies from Greater Paris, the US, and Chile, which reported 90-day survival rates ranging from 46 to 65%. A recent comprehensive analysis confirmed these results, indicating a hospital discharge survival rate of 62.9% [2]. Survival rates depend on various factors, including the distribution of local resources, criteria for selecting patients, timing of ECMO initiation, and the expertise of the treatment centers. Our study population predominantly consisted of younger patients with few or no significant comorbidities. By deviating from the standardized protocols for ARDS rescue therapy and applying modified criteria for ECMO in patients with severe ARDS, we observed a considerable improvement in outcomes [17]. This underscores the importance of careful patient selection to enhance outcomes for patients with ARDS on ECMO support. In addition, in our center, an early intervention strategy with ECMO support contributed to our promising results. The normal pH and lactic acid levels observed prior to ECMO cannulation, alongside the early initiation of ECMO support within 12 h of starting ventilation therapy, affirm our institution’s proactive ECMO strategy during ICU management. In patients who require VV ECMO, it is crucial to closely monitor their progress in the ICU and quickly identify the transition point when ECMO support becomes necessary. Accordingly, the implementation of ECMO should be tailored to individual cases, with a thoughtful assessment of the expected benefits and potential risks.

In our study, young age was a significant perioperative factor associated with better early outcomes in patients with ARDS receiving ECMO support. Previous studies have also demonstrated a strong association between older age and higher rates of mortality and complications, supporting the results of our study [18]. Consequently, older age can be considered an independent factor contributing to poorer early outcomes. Conversely, another study acknowledged that, while higher mortality rates were observed in older patients with ARDS, this correlated statistically with a higher rate of treatment withdrawal among this demographic. These findings suggest that age may influence mortality rates by influencing treatment withdrawal [19]. Our results, indicating a relationship between the age of patients and mortality rates, stem from correlation analysis and do not establish causality. Therefore, further research, including longitudinal studies, is warranted to explore and establish causality between age and early outcomes.

We found rehabilitation therapy to be another crucial perioperative factor related to improved early outcomes in patients with ARDS supported by ECMO. This finding underscores the feasibility, safety, and effectiveness of awakening patients and engaging them in rehabilitation therapy while on ECMO, particularly in the context of COVID-19. Initiating early rehabilitation therapy involves encouraging patients to walk early, minimizing the use of sedatives to maintain consciousness, and promoting extubation. These prerequisites for patients undergoing rehabilitation therapy likely contribute to the efficacy of the ongoing ECMO treatment. According to several studies, patients with respiratory failure due to COVID-19 who received active rehabilitation therapy while on awake ECMO demonstrated significant functional improvements [20,21,22]. Similarly, better outcomes were observed in patients who underwent tracheostomy, which could be attributed to their consciousness and ability to participate actively in rehabilitation therapy [23]. Rehabilitation therapy for patients on ECMO was always conducted with close monitoring of vital signs by the ECMO team. Such safe and active implementation of rehabilitation therapy, leading to positive patient outcomes, necessitates effective co-operation among the medical staff, similar to our center’s approach.

Among the patients who received ECMO, those with COVID-19 were treated in isolated rooms. Our study results showed that the incidence of bleeding during anticoagulation therapy while on ECMO was not high, and even when bleeding occurred, it did not lead to death or other adverse outcomes, confirming that ECMO can be safe and effective even in an isolated environment. In our study, most patients underwent anticoagulation therapy during the ECMO period, and only two patients experienced bleeding complications related to anticoagulation therapy. These patients discontinued anticoagulation therapy, were successfully weaned off ECMO, and were discharged. Given that bleeding in an isolated environment may not be detected as quickly as in other patients, posing a potential risk, it is still important to closely monitor for signs of bleeding in patients with COVID-19 undergoing ECMO.

The administration of immunosuppressive therapy, regardless of timing, was linked to failure to wean from ECMO. Immunosuppressant use increases the likelihood of secondary infections. However, our findings indicate that secondary infections following immunosuppressive therapy did not influence the weaning process from ECMO. It is conceivable that within our treatment strategy for COVID-19, immunosuppressive therapies were employed as a final resort for patients who did not show improvement with other interventions, thus correlating with poorer outcomes in this particular patient population.

Our study is subject to several limitations that should be taken into account. First, its single-center design may limit the generalizability of the findings to other settings that differ in resource availability. Due to the nature of this study being conducted at a single institution, the number of COVID-19-diagnosed patients who received ECMO treatment was relatively low. This limitation is primarily due to our institution’s selective criteria for VV ECMO and the high rate of insurance denial in South Korea [24]. Second, the retrospective nature of this study may introduce inherent biases, including selection and recall bias. Although this is a single-center study, its multidisciplinary approach and incorporation of early aggressive rehabilitation therapy represent a significant strength, including a large sample size from a nationally designated COVID-19 treatment hospital. Identifying the key factors influencing the early outcomes of patients with COVID-19 treated with ECMO highlights the significance of our findings. Additional longitudinal and prospective studies are necessary to elucidate the factors affecting early outcomes in patients with COVID-19 undergoing ECMO. Moreover, given that these outcomes are influenced not by a single factor but by the interplay of multiple factors, multivariate analysis will be necessary for a holistic interpretation of these results.

## 5. Conclusions

Our study has demonstrated a high success rate in weaning patients with COVID-19 off ECMO and achieving significant survival to discharge despite the challenges of extreme isolation required for these patients. Factors such as younger age and the implementation of rehabilitation therapies have been significantly associated with better early outcomes. These findings underscore the importance of a multidisciplinary approach to care, highlighting the potential of targeted rehabilitation interventions and the benefits of ECMO in managing severe respiratory failure in younger patients with COVID-19.

## Figures and Tables

**Table 1:** Patient characteristics.

Variables			N = 25
Age			51.88 (15.38)
BMI			27.69 (4.98)
Male			14 (56%)
Presence of smoking history			3 (12%)
Presence of underlying disease			13 (52%)
SOFA score			5.60 (1.83)
Blood test results before ECMO application	pH		7.38 (0.07)
	Lactic acid (mmol/L)		1.20 [1.00, 1.90]
PaO_2_/FiO_2_ ratio			78.68 (24.22)
Length of ventilator support before ECMO (hours)			12.00 [5.00, 25.00]
Application of prone position			8 (32%)
Usage of medication	Systemic steroid	Before ECMO	18 (72%)
		After ECMO	7 (28%)
	Antiviral agents		14 (56%)
	Immunosuppressive therapy		12 (48%)
	Inotropic and or vasopressor		18 (72%)
	Muscle relaxant		23 (92%)
Anticoagulation method (%)	Maintain from initiation of ECMO		15 (60.0)
	Anticoagulant change		6 (24.0)
	Termination due to bleeding complications		2 (8.0)
	No anticoagulation		2 (8.0)
Awakening ECMO			7 (28%)
ECMO (%)	Maintain		19 (76.0)
	Circuit change		4 (16.0)
	Mode change		2 (8.0)
Length of ECMO support (days)			14.88 (10.05)
Complication			11 (44%)
Rehabilitation			16 (64%)

BMI, body mass index; SOFA, Sequential Organ Failure Assessment; ECMO, extracorporeal membrane oxygenation. Values are presented as the mean (standard deviation), median (range), or number (%).

**Table 2:** Factors associated with ECMO weaning success of patients with COVID-19.

Variables			Weaning Success (n = 18)	Weaning Failure (n = 7)	*p*-Value
Age			46.78 (15.06)	65.00 (5.03)	<0.001
BMI			28.41 (5.41)	25.82 (3.28)	0.162
Presence of smoking history			1 (5.6%)	2 (28.6%)	0.180
Presence of underlying disease			8 (44.4%)	4 (57.1%)	0.673
SOFA score			5.28 (1.74)	6.43 (1.90)	0.194
Blood test results before ECMO application	pH		7.38 (0.08)	7.36 (0.06)	0.505
	Lactic acid (mmol/L)		1.35 [1.00, 1.90]	1.20 [0.98, 2.25]	>0.999
PaO_2_/FiO_2_ ratio			76.83 (25.75)	83.43 (20.79)	0.518
Length of ventilator support before ECMO (hours)			10.50 [5.00, 24.75]	24.00 [5.50, 36.00]	0.832
Application of prone position			5 (27.8%)	3 (42.9%)	5 (27.8)
Usage of medication	Systemic steroid	Before ECMO	13 (72.2%)	5 (71.4%)	>0.999
		After ECMO	5 (27.8%)	2 (28.6%)	
	Antiviral agents		10 (55.6%)	4 (57.1%)	>0.999
	Immunosuppressive therapy		6 (33.3%)	6 (85.7%)	0.030
	Inotropic and or vasopressor		13 (72.2%)	5 (71.4%)	>0.999
	Muscle relaxant		17 (94.4)	6 (85.7)	0.490
Anticoagulation method (%)	Maintain from initiation of ECMO		12 (66.7)	3 (42.9)	0.365
	Anticoagulant change		3 (16.7)	3 (42.9)	
	Termination due to bleeding complications		2 (11.1)	0 (0.0)	
	No anticoagulation		1 (5.6)	1 (14.3)	
Awakening ECMO			7 (38.9%)	0 (0.0%)	0.133
ECMO (%)	Maintain		13 (68.4)	6 (31.6)	0.340
	Circuit change		4 (100.0)	0 (0.0)	
	Mode change		1 (50.0)	1 (50.0)	
Length of ECMO support (days)			14.06 (9.85)	17.00 (11.03)	0.551
Complication			7 (38.9)	4 (57.1)	0.656
Rehabilitation			16 (88.9%)	0 (0.0%)	<0.001

BMI, body mass index; SOFA, Sequential Organ Failure Assessment; ECMO, extracorporeal membrane oxygenation. Values are presented as the mean (standard deviation), median (range), or number (%).

**Table 3:** Association between secondary infection following immunosuppressive treatment and success in weaning off ECMO.

		Immunosuppressive Treatment (n = 12)	Weaning Success	Weaning Failure	*p*-Value
Secondary infection (%)	Yes	5 (41.7)	3 (25.0)	2 (16.7)	0.558
	No	7 (58.3)	3 (25.0)	4 (33.3)	

ECMO, extracorporeal membrane oxygenation. Values are presented as numbers (%).

**Table 4:** Factors associated with in-hospital mortality of patients with COVID-19.

Variables			Discharge (n = 17)	Death (n = 8)	*p*-Value
Age			45.38 (15.32)	63.44 (5.90)	<0.001
BMI			28.94 (5.43)	25.45 (3.22)	0.055
Presence of smoking history			1 (6.2%)	2 (22.2%)	0.530
Presence of underlying disease			4 (57.1%)	8 (44.4%)	0.673
SOFA score			5.19 (1.68)	6.33 (1.94)	0.158
Blood test results before ECMO application	pH		7.38 (0.08)	7.37 (0.06)	0.756
	Lactic acid (mmol/L)		1.20 [1.00, 1.90]	1.20 [1.05, 2.05]	0.915
PaO_2_/FiO_2_ ratio			73.06 (18.24)	88.67 (31.00)	0.194
Length of ventilator support before ECMO (hours)			8.50 [4.75, 18.75]	24.00 [6.00, 48.00]	0.222
Application of prone position			4 (25.0%)	4 (44.4%)	0.394
Usage of medication	Systemic steroid	Before ECMO	11 (68.8%)	7 (77.8%)	>0.999
		After ECMO	5 (31.2%)	2 (22.2%)	
	Antiviral agents		10 (62.5%)	4 (44.4%)	0.434
	Immunosuppressive therapy		6 (37.5%)	6 (66.7%)	0.226
	Inotropic and or vasopressor		11 (68.8%)	7 (77.8%)	>0.999
	Muscle relaxant		15 (93.8)	8 (88.9)	>0.999
Anticoagulation method (%)	Maintain from initiation of ECMO		12 (70.6)	3 (37.5)	0.136
	Anticoagulant change		2 (11.8)	0 (0.0)	
	Termination due to bleeding complications		2 (11.8)	4 (50.0)	
	No anticoagulation		1 (5.9)	1 (12.5)	
Awakening ECMO			0 (0.0%)	7 (38.9%)	0.133
ECMO (%)	Maintain		12 (63.2)	7 (36.8)	0.824
	Circuit change		3 (75.0)	1 (25.0)	
	Mode change		1 (50.0)	1 (50.0)	
Length of ECMO support (days)			17.00 (11.03)	14.06 (9.85)	0.551
Complication			6 (66.7)	5 (31.2)	0.115
Rehabilitation			16 (88.9%)	0 (0.0%)	<0.001

BMI, body mass index; SOFA, Sequential Organ Failure Assessment; ECMO, extracorporeal membrane oxygenation. Values are presented as the mean (standard deviation), median (range), or number (%).

## Data Availability

The raw data supporting the conclusions of this article will be made available by the authors upon request.

## Supplementary Materials

The following supporting information can be downloaded at: https://www.mdpi.com/article/10.3390/jcm13195922/s1, Table S1: comorbidities associated with ECMO weaning success of patients with COVID-19; Table S2: comorbidities associated with in-hospital mortality of patients with COVID-19.
