# Supplementary material for: Factors Associated with Outcomes of Patients with Veno-Venous Extracorporeal Membrane Oxygenation for COVID-19
Source: J Clin Med. 2024 Oct 4;13(19):5922. doi: 10.3390/jcm13195922 (PMC11478145; doi:10.3390/jcm13195922)
Supplement: Supplementary file 1 [file jcm-13-05922-s001.zip › jcm-3217436-supplementary.pdf]

Supplementary Table S1. Comorbidities associated with ECMO weaning success of patients with COVID-19

Variables		Weaning success (n = 18)	Weaning failure (n = 7)	*P*-value
Comorbidity (%)	None	3 (37.5)	10 (58.8)	0.176
	HTN	1 (12.5)	3 (17.6)	
	DM	1 (12.5)	2 (11.8)	
	DM & HTN	0 (0.0)	2 (11.8)	
	Cancer	2 (25.0)	0 (0.0)	
	Thromboembolism	1 (12.5)	0 (0.0)	

ECMO, extracorporeal membrane oxygenation; HTN, hypertension; DM, diabetes mellitus.

Values are presented as the mean (standard deviation), median [range], or number (%).

Supplementary Table S2. Comorbidities associated with in-hospital mortality of patients with COVID-19

Variables		Discharge (n = 17)	Death (n = 8)	*P*-value
Comorbidity (%)	None	10 (55.6)	3 (42.9)	0.594
	HTN	3 (16.7)	1 (14.3)	
	DM	2 (11.1)	1 (14.3)	
	DM & HTN	2 (11.1)	0 (0.0)	
	Cancer	1 (5.6)	1 (14.3)	
	Thromboembolism	0 (0.0)	1 (14.3)	

ECMO, extracorporeal membrane oxygenation; HTN, hypertension; DM, diabetes mellitus.

Values are presented as the mean (standard deviation), median [range], or number (%).
